# Extracellular Vesicles with Possible Roles in Gut Intestinal Tract Homeostasis and IBD

**DOI:** 10.1155/2020/1945832

**Published:** 2020-01-13

**Authors:** Xin Chang, Shu-Ling Wang, Sheng-Bing Zhao, Yi-Hai Shi, Peng Pan, Lun Gu, Jun Yao, Zhao-Shen Li, Yu Bai

**Affiliations:** ^1^Department of Gastroenterology, Gongli Hospital, The Second Military Medical University, Shanghai, China; ^2^Department of Gastroenterology, Changhai Hospital, Second Military Medical University/Naval Medical University, Shanghai, China; ^3^Department of Gastroenterology, The Second Clinical Medical College, Jinan University, Shenzhen, China

## Abstract

The intestinal tract consists of various types of cells, such as epithelial cells, Paneth cells, macrophages, and lymphocytes, which constitute the intestinal immune system and play a significant role in maintaining intestinal homeostasis by producing antimicrobial materials and controlling the host-commensal balance. Various studies have found that the dysfunction of intestinal homeostasis contributes to the pathogenesis of inflammatory bowel disease (IBD). As a novel mediator, extracellular vesicles (EVs) have been recognized as effective communicators, not only between cells but also between cells and the organism. In recent years, EVs have been regarded as vital characters for dysregulated homeostasis and IBD in either the etiology or the pathology of intestinal inflammation. Here, we review recent studies on EVs associated with intestinal homeostasis and IBD and discuss their source, cargo, and origin, as well as their therapeutic effects on IBD, which mainly include artificial nanoparticles and EVs derived from microorganisms.

## 1. Introduction

The homeostasis of the intestinal tract is the most complex homeostasis within the human body due to the direct exposure to the digestive residue, millions of pathogens, and high concentrations of foreign antigens [1]. During this process, the intestinal mucosal barrier plays a pivotal role in detecting and clearing the pathogenic microbial debris, while maintaining a peaceful coexistence with them. As for the intestinal defense system, it mainly consists of three parts, including the mucus layer, intestinal epithelial cells (IECs), and other immune cells, such as lymphocytes and macrophages that are associated with the innate immune system. Additionally, effective communication among these cells plays a critical role in maintaining the intestinal homeostasis, which is mainly mediated by extracellular factors and receptors, such as growth factor and its receptor tyrosine kinase [2, 3]. However, in recent decades, extracellular vesicles (EVs) have been recognized as a novel mediator not only for the cell-to-cell but also for the organism-to-cell interaction [4–6]. In addition, the mammalian intestine encounters about 10 trillion (10^13^) microbes which is approximately equal to ten times the number of our total cells, and the whole genome from this microorganism even exceeds that of the entire human genome by 150- to 400-fold [7]. As a result, the coexistence with gut microbiota plays a significant role in maintaining intestinal homeostasis, which has been recognized as a major determinant to our health [8, 9].

Microbiota-derived EVs carry a large diversity of compounds that can affect various pathways in the host. Emerging evidence has demonstrated the role of EVs in bacterial survival and host interaction [6]. EVs are submicron-circulating vesicles found in all bodily fluids and in all species, including bacteria. Eukaryotic cells' EVs originate from the process of plasma membrane budding or fusion of multivesicular endosomes with the plasma membrane. Relatively, EVs derived from Gram-positive and Gram-negative bacteria may disperse in extracellular space by outward budding of the prokaryotic membrane [10–12]. In past reviews, the EVs tend to be divided into three main subsets known as exosomes, microvesicles (MVs), and apoptotic bodies [13, 14]. Their intrinsic heterogeneity can separate and characterize them with varying sizes, molecular patterns, and triggering mechanisms. Exosomes (40-150 nm) are produced via a lyso-endosomal system. MVs (100-1000 nm) are generated through the direct budding of the cell plasma membrane in a calcium-dependent process. Apoptotic bodies (>2000 nm) are released during cell apoptosis and are the most heterogeneous type, with a diverse morphology. However, this classification neglects bacteria-released membrane vesicles (20-400 nm) which are regarded as MVs or outer membrane vesicles (OMVs) based on whether they are Gram-negative or Gram-positive [15, 16]. In this review, bacteria-released membrane vesicles were classified as EVs due to the mechanism for any organisms' intercellular communication. Therefore, EVs are evolutionarily conserved across eukaryotes, bacteria, and archaea. Here, we highlight specific paradigms of cell-to-cell and organism-to-cell communication in intestinal homeostasis. Additionally, we provide a brief update on the clinical application of EVs as delivery vehicles as well as the sources of diagnostic markers.

### 1.1. EVs

EVs are found in most physiological fluids, including urine, breast milk, and bile [17]. Additionally, EVs can also be collected from cell culture supernatant. EVs within the intestinal tract can be derived from cells, organisms, or physiological fluids, such as succus entericus. A previous study found that patients with malignant common bile stenoses contained significantly higher concentrations of EVs than healthy controls in bile samples, indicating that characteristics of EVs vary at different states of the body [18]. Furthermore, EVs contain bioactive cargo, such as nucleic acids (DNA, mRNA, microRNA, and other noncoding RNAs), proteins (receptors, transcription factors, enzymes, and extracellular matrix proteins), and lipids which can regulate the functions of the recipient cell [19–21].

### 1.2. Classification of EVs

**Figure 1:**
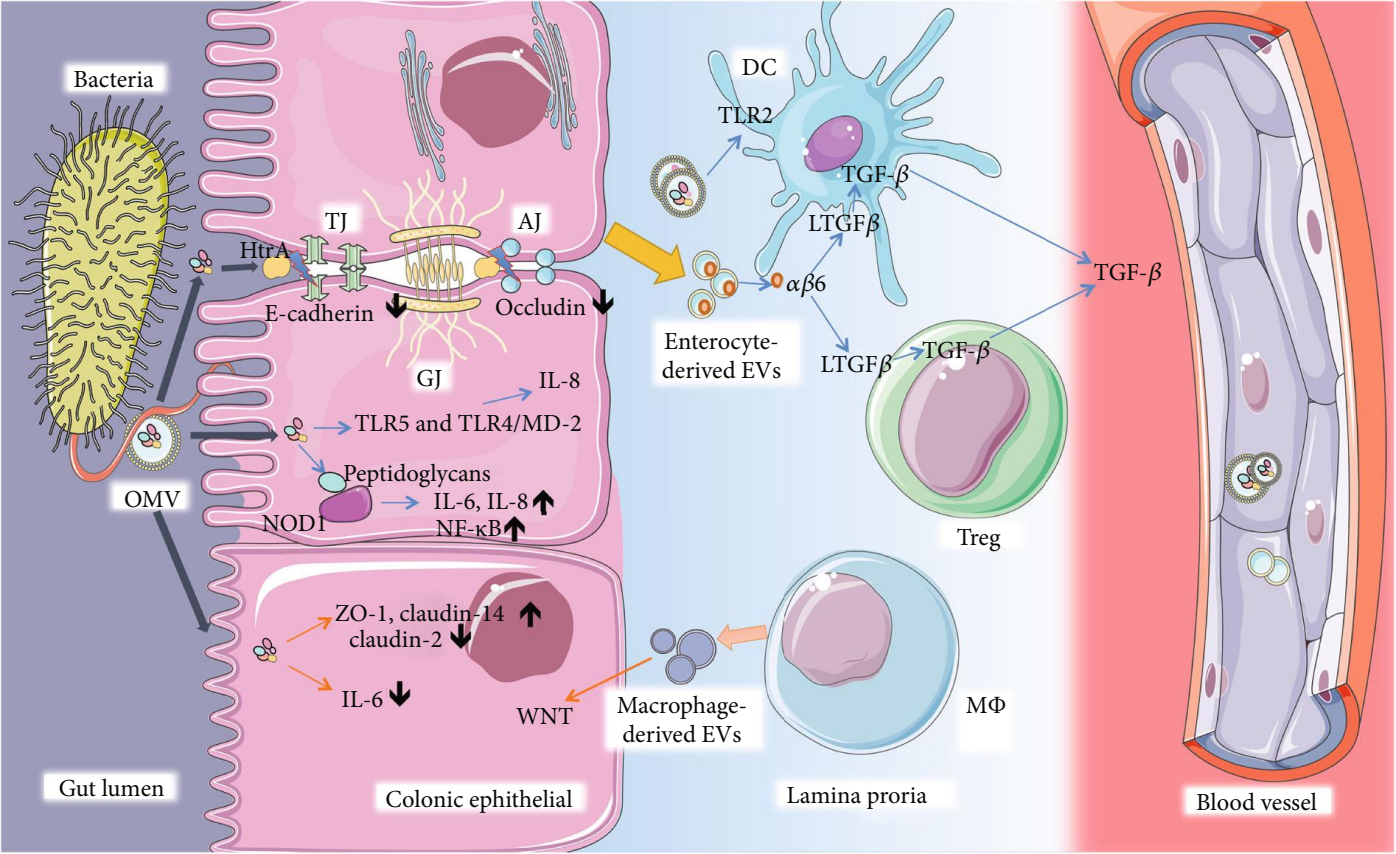
Classification of extracellular vesicles according to the mechanism of generation. Extracellular vesicles include exosomes, microvesicles, apoptotic bodies, out membrane vesicles, and membrane vesicles (not shown in the figure) in this review. Exosomes are produced by budding from multivesicular bodies. Microvesicles are generated intracellularly from the extracellular membrane. Apoptotic bodies are originated upon cell fragmentation during apoptotic cell death.

Based on present studies regarding the biogenesis and size of EVs, three categories of EVs as well as several terms, including microvesicles, exosomes, ectosomes, oncosomes, and outer membrane vesicles, are presented [22]. Exosomes refer to EVs ranging between 40 and 150 nm in diameter and are produced from the multivesicular endosome pathway. While those in the range of 100 to 1000 nm are microvesicles or microparticles derived from plasma membrane. Microvesicles that separated at approximately 10 to 14,000 g are heterogeneous. In contrast, microvesicles separated at 100,000 g are homogeneous [17, 23–25]. Apoptotic bodies with large populations originate from membrane blebbing and cellular disassembly from cell fragmentation when the cytoskeleton breaks at the beginning of apoptosis. Recently, larger-size EV subpopulations (1-10 *μ*m diameter) were distinguished from highly migratory cancer cells and were termed as oncosomes due to their distinguishing biomolecules and unique extraction methods (Figure 1). As mentioned above, the present classification is based on the eukaryote system excluding the bacteria-released membrane vesicles. However, the shedding of membrane vesicles is ubiquitous in bacteria. The production of OMVs was first discovered in Gram-negative bacteria in 1963 [26]. They were identified as OMVs due to them originating from the controlled blebbing of the outer membrane of Gram-negative bacteria. Moreover, recent work has shown the vesicles and MVs of bacteria refer to those of archaea and Gram-positive bacterial origin [6, 27]. OMVs refer to those originating from Gram-negative bacteria with a diameter of about 20-400 nm, while MVs are cytoplasmic membranes of Gram-positive bacteria with a diameter typically of 20–150 nm [28]. Both of OMVs and MVs can carry DNA, sRNA, proteins, and other factors to the recipient cells [29].

### 1.3. Biogenesis and Characteristics of EVs

Exosomes originate through the lyso-endosome pathway. Exosomes are released upon the fusion of multivesicular bodies (MVBs) with the plasma membrane. MVBs are vesicular entities generated in the maturation process of the early endosomes formed by plasma membrane invagination. Within the cytoplasm, the membrane of MVBs forms intraluminal vesicles (ILVs) by inward budding. After MVBs fuse with the plasma membrane, they release inside ILVs, which are called exosomes [19, 30, 31]. According to the International Society for Extracellular Vesicles (ISEV), there are minimal requirements to claim the presence of exosome isolation; several experiments need to be conducted to characterize the existence of the exosomes, such as electron microscopy, concentration-monitoring techniques, and western blotting [14]. The cup-shaped lipoidal vesicle structure is the typical feature of EVs under the electron microscope. In biochemistry, the tetraspanin superfamily was previously thought to be a specific marker of exosomes. However, MV has also been reported to bear CD63, CD9, and CD81 tetraspanin proteins in recent years. In multiple studies, investigations concerning Alix and TSG101 involving the exosome forming process and heat shock proteins HSC70 and HSP90 have also been carried out with exosomes [32].

According to previous studies, microvesicles are mainly derived from plasma via a calcium-regulated pathway which requires lipid formation for budding out [33]. As mentioned above, apart from exosomes, MVs also contain the tetraspanin protein family (CD9, CD63, and CD81), thus indicating the significance of these proteins in the budding and fusion of the membrane [34]. Moreover, MVs can also generate a more heterogeneous subpopulation of extracellular vesicles carrying surface markers and receptors from their parental cell, which takes part in intercellular communication and capacitates their identification in the laboratory [35]. The studies included in this review do not discriminate endosome-derived from plasma membrane-derived EVs. In this review, we use the term “EVs” rather than the term in the cited literature, thereby no longer distinguishing an endosomal or plasma membrane origin.

As a result, these tetraspanin proteins, including CD9, CD63, and CD81, were regarded as markers to evaluate the purity of the molecules after isolation. Additionally, physical properties, such as particle size, were also characteristic during the process of isolation via ultracentrifugation, density gradient separation, and polymer-based precipitation methods.

## 2. EVs in IBD

### 2.1. Microbiota-Derived EVs

EVs are produced by all domains of life, including microorganism Gram-negative and Gram-positive bacteria, archaea, fungi, and protozoa [28, 36]. The alterations in microbiota colonizing intestines have been implicated in the pathogenesis and development of many diseases and particularly in IBD [37, 38]. The balance between host and commensal microbe in the intestine is the key to maintaining a healthy human state, as they can regulate the maturation and functions of IECs and various immune cells. EVs released from both pathogenic and commensal bacteria are important regulators of host-pathogen communication that regulate immunomodulation and the corresponding signaling pathways. For instance, *Pseudomonas aeruginosa* OMV-mediated short RNAs (sRNAs) reduced the secretion of IL-8 in IECs which were induced by lipopolysaccharide (LPS). The enriched sRNA52320 can attenuate OMV-induced KC cytokine secretion and neutrophil infiltration [39]. On the contrary, the EVs derived from the physiological fluids may also influence the intestinal microbiota. A previous study used EVs from the sera of Toll-like receptor (TLR) 2 knockout mice and wild-type mice to interact with *Lactobacillus* or *Bifidobacterium* which are common bacteria in the gut. The study found that EVs significantly reduced the activity of TLR2/6 both in *Bifidobacterium* and *Lactobacillus*, thus contributing to the aggregation of pathogens [40]. EVs were first discovered over 40 years ago. In 1967, Chatterjee and Das revealed the excretion of cell wall material in *Vibrio cholera* by electron microscopy. They found that *Neisseria meningitides* released endotoxins in the form of cell wall blebs in vivo [41]. EVs produced by commensal bacteria in the gastrointestinal tract of animals are distributed throughout the gut lumen with a variety of biomolecules, nucleic acids, enzymes, toxins, and metabolites. The engagement of extracellular products from commensal bacteria in immunomodulatory activities has been noted since 1967 [41]. However, the mechanism involved has not yet been studied completely or systematically.

Sometimes, microbiota-derived EVs serve as bad factors in digestive tract homeostasis. *Helicobacter pylori* (Hp) infection can lead to gastritis, ulceration, or malignancy due to a degree of adhesion to the epithelium. Furthermore, in 2003, Ismail et al. revealed that there is no need for Hp to directly contact the epithelium cell to cause gastritis and that OMVs from Hp could be accepted by the host cells and further stimulate various responses independently [42]. Recently, EVs from enterohemorrhagic *Escherichia coli* (EHEC) O157 during growth were found to stimulate the production of interleukin-8 (IL-8) in IECs via the TLR5 and TLR4/MD-2 complex signaling pathway [43]. They also deliver the hemolysin from EHEC to microvascular endothelial cells and mitochondria, thus triggering apoptosis [44]. OMVs from bacteria are the cargo of many various ligands of pattern recognition receptors (PRR), including DNA, RNA, lipoproteins, LPS, and peptidoglycan, which initiate proinflammatory signaling cascades. OMVs from commensal *Escherichia coli* containing peptidoglycans that can colonialize with Nucleotide Binding Oligomerization Domain Containing 1 (NOD1), trigger the NOD1 signaling pathway, and improve the expression of NF-*κ*B, IL-6, and IL-8 [45]. In addition, OMVs can enter the IECs via clathrin-dependent endocytosis and give rise to DNA damage [46]. In dextran sulfate sodium- (DSS-) induced colitis, the gut microbiota regulate intestinal UDP-glucuronosyltransferase 1A1 (UGT1A1) through secreting cargo that can interact with epithelial cells directly [47]. *Vibrio cholera* secrets EV-associated Zn-dependent hemagglutinin protease (HAP), and cholera toxins are transported to human IECs to induce dose-dependent apoptosis [48, 49]. Furthermore, these EVs internalized by IECs induce the expression of IL-8, GM-CSF, and chemokines, such as CCL2, CCL20, and thymic stromal lymphopoietin, in epithelial cells by activating the MAPK and NF-*κ*B pathways in a NOD1-dependent manner [50].

In addition, microbiota-derived EVs may help to maintain the homeostasis of the intestinal tract. It is generally known that the integrity of the gastrointestinal epithelial layer, consisting of the physical and biochemical barrier, is critical in fighting against various toxins and pathogens. Apart from these cells, intestinal microbes, especially probiotic bacteria, can modulate barrier integrity by reducing gut epithelial proinflammation, reinforcing tight junctions, and other reciprocal interactions among commensal bacteria, the epithelium, and the mucosal immune system. *Escherichia coli* C25, the first colonized bacteria in the intestine, elicit a mild proinflammatory effect on host epithelial cells with upregulated TLR in vitro, which is considered to be the mediator of a rapid but more controllable reaction to pathogenic bacteria in vivo [51]. Probiotic *Escherichia coli* Nissle 1917 (EcN) act as beneficial colonizers in the human gut by secreting the protein TcpC to regulate the expression of tight junction protein in IBD [52]. However, the independence of TcpC has been verified in probiotic *E. coli*-derived EVs. In 2016, Alvarez et al. illustrated that EVs from both EcN and ECOR63 have a strengthening ability based on TcpC. EVs isolated from these probiotics can promote the upregulation of ZO-1 and claudin-14 and downregulation of claudin-2, thus helping the reinforcement of the epithelial barrier, while the specific mechanism has not yet been illustrated [53]. EVs from *Bacteroides thetaiotaomicron* (BtMinpp) may protect enzymes from degradation by gastrointestinal proteases and promote intracellular Ca^(2+)^ signaling, thus maintaining the physiological responses of the digestive system [54]. Meanwhile, EVs isolated from intestinal microbiota have been evaluated in an experimental IBD model. Owing to the complexity of the gut microbiota, their roles are different: EVs from *E. coli* induce colon epithelial cells to release the proinflammatory cytokine IL-6, while *Akkermansia muciniphila* can alleviate this. The oral application of EVs from *A. muciniphila* ameliorates the levels of inflammation both in LPS-stimulated macrophages and IECs [55].

However, pathogenic EVs can disrupt intestinal barrier integrity and exaggerate the invasion of harmful components into the submucosa, thus contributing to the pathogenesis of IBD. *Campylobacter jejuni* has been detected in many tissues, such as lamina propria, and blood. Recently, *C. jejuni* was reported to cleave cell-to-cell junction factors, such as E-cadherin, and occlude facilitating the invasion of pathogens into IECs via serine protease HtrA and bacterial EVs [56, 57]. The toxicity of HtrA proteins and their orthologues are nonnegligible in both prokaryotes and eukaryotes [58]. The function of E-cadherin to establish and maintain epithelial integrity has been discussed in many studies [59, 60]. Deleting the HtrA protein in *C. jejuni* can alter E-cadherin shedding [61]. Furthermore, pretreatment with methyl-beta-cyclodextrin partially blocks OMV-induced host immune responses, demonstrating the effect of lipid rafts on host cell plasma membranes during interactions with *C. jejuni* OMVs [62].

### 2.2. Enterocyte-Derived EVs

The essential function of the intestinal epithelium is to form a barrier regulating the interactions with luminal contents. It can also act as the underlying immune system, regulating the inflammation response. Through complex communication with the pathogens and the immune system, IECs maintain intestinal homeostasis.

### 2.3. EVs Derived from IEC Regulation of Gut Immune Cells

IECs promote the development of dendritic cells (DCs) and macrophages with tolerogenic properties by producing numerous immunoregulatory signals, including TGF-*β*, thymic stromal lymphopoietin (TSLP), and retinoic acid [63–65]. Professional antigen-presenting cells (APC) have been verified to secrete major histocompatibility complex- (MHC-) bearing vesicles called exosomes, which are a subset of EVs [66]. Although IECs are not primarily APCs, they constitutively express MHC I, MHC II, and HLA-DM localized in vesicular structures from biopsies and HT-29 cells [67]. Additionally, EVs from these enterocyte cells can be released from either the apical or basolateral side. They preferentially interact with DCs and potentiate antigen-presenting capacity [68]. The fact that IECs release EVs has been known for more than ten years, and this investigation complemented the lack of direct contact between IEC and CD4+ T-cells [69]. Their EVs express immunomodulatory molecules, such as major histocompatibility complex (MHC) class I and class II molecules, whose expression levels are much higher in inflammatory conditions compared with basal conditions [68–70]. MHC II is essential in initiating adaptive immunity; its upregulation during B-cell development suggests its role in consolidating B-cell maturation [71]. The adaptive immune response is related to the high expression of MHC I molecules in esophageal adenocarcinoma development [72]. Except for the normal antigen-presenting molecules enriched on EV surfaces, EVs derived from IECs specifically display A33 antigens used to identify the origin of the EVs [67, 68, 73]. EVs derived from IECs have been demonstrated to be necessary for tolerogenic immune cells and directing appropriate innate and adaptive immune cell responses in both physiology and pathological states. Additionally, tolerogenic DCs are considered indispensable for maintaining intestinal homeostasis [1]. EVs derived from IECs carrying *αβ*6 activate LTGF*β* in intestinal tolerogenic DCs and Tregs, which first produces TGF-*β*. After internalizing the EVs, DCs improve the expression of TGF-*β* and finally induce the Treg cell and drive tolerogenic responses [74]. Epithelial EVs may participate in this tolerogenic process directly. In 2001, Karlsson et al. named exosome-like structures as tolerosomes, which were isolated from rat IECs and can induce antigen-specific tolerance when administered to naive recipient rats intraperitoneally [75]. In 2016, Jiang et al. demonstrated that EVs originate from IECs containing TGF-*β* inhabited CD4+ cell proliferation under physiological conditions [76]. In posttrauma immune dysfunction, the expression of CD63 (a specific marker of exosome) and the epithelial cell-specific marker epithelial cell adhesion molecule (EpCAM) were improved greatly, illustrating that EVs from IECs induce DC apoptosis, suppress DC maturation, and inhibit the Ag-presenting function of DCs [77]. The EpCAM induces the homophilic interaction molecule between IECs and intraepithelial lymphocytes in the physical mucosal epithelium and regulates the positive effect of EVs on the intestinal tract immune balance [76, 78].

### 2.4. IEC-Derived EVs Promoting Repairment and Regulating the Inflammatory Response

EVs originated from IECs carry the component promoting epithelial healing, coinciding with the resolution of inflammation. Annexin A1 (ANXA1) facilitates the repair of intestinal mucosal wounds in a murine model of colitis, and their release is elevated during wound closure [79, 80]. In 2015, Giovanna et al. reported that EVs derived from IECs containing ANAXA1 can be used to activate wound repair circuits and promote epithelial restitution. During mucosal repair, ANAXA1 in EVs acted as an endogenous mediator of wound healing by binding to formyl peptide receptors (FPRs) expressed on responsive cells [81]. In 2018, Zhang et al. identified that EVs isolated from the mucosal-luminal interface of IBD patients contained defense protein MPO [82]. The MPO function is to induce the oxidation reaction by producing reactive oxidants, such as hypohalous acids [83–85]. The increased level of oxidative stress can withstand the microbes in the gut of patients with IBD [86]. However, EVs isolated from the intestinal lumen fluid of patients with IBD had a proinflammatory effect on IECs in vitro [87]. This discrepancy may be caused by the source of the EVs. This is because intestinal lumen fluid is quite different from the aspirate of the mucosal-luminal interface. The alteration of enterobacteria has already been linked with gut-associated inflammation, which is itself a crucial risk factor for colon cancer. In 2015, Deng et al. revealed that enterotoxigenic *Bacteroides fragilis* secreted EVs that could induce the production of intestinal mucosa-derived EVs containing elevated levels of sphingosine-1-phosphate, CCL20, and prostaglandin E2 [88]. Additionally, CCL20 and prostaglandin E2 recruit Th17 cells through the MyD88-mediated pathway [88]. Several studies have demonstrated the role of sphingosine-1-phosphate in tumorigenesis [89–91]. These studies also implicated a possible role of EVs derived from normal intestinal mucosa in suppression of CCL20 and other proinflammatory cytokines [88].

### 2.5. Immune Cell-Derived EVs

Previous studies have proven the link between the abnormal immune responses and IBD. Both the innate and adaptive immune responses contribute greatly to the IBD pathogenesis. The innate immune responses act faster to trigger the phagocytic responses and antigen presentation, along with initiating the adaptive immune system. These involve various immune cells, such as the macrophages, DCs, neutrophils, and monocytes. Several studies have shown the immune-stimulatory effects of the EVs from DCs [92]. The inhibition of T-cell proliferation by EVs derived from DCs has been proposed to play a key role in suppression of the inflammation-related disease, such as IBD [93, 94]. As compared to the nongene-modified BMDC, TGF-*β*1 gene-modified BMDC can lead to the release of immunosuppressive EVs that contain high levels of TGF-*β*1 and elicit stronger inhibitory effects on the T-cell proliferation [95]. In addition, much work has demonstrated EVs from conditioned DC might promote IBD in remission. The EVs derived from DCs treated with *S. japonicum*-soluble egg antigens or IL-10 play a protective role during acute IBD development [94, 96, 97]. Furthermore, EVs from other immune cell can influence disease progression in different ways. Intestinal mucosa polymorphonuclear neutrophil (PMN) infiltration is common in IBD. During the infiltration of these immune cells, myeloperoxidase (MPO) can be released into the extracellular environment. The MPO release is common in acute and chronic inflammation. During the progression of IBD, MPO can damage the gut barrier. In 2019, Thomas et al. explored a new regulation mechanism between MPO and PMNs during inflammation [98]. With the help of EVs, MPO can be protected and delivered to IECs. The tissue-infiltrating PMNs together with MPO enhanced the inflammatory response and inhibited the wound closure through the regulation of the IEC migration and proliferation [98]. Similarly, Butin-Israeli et al. confirmed the role of EVs armed with the proinflammatory microRNAs in mediating the accumulation of the double-strand breaks (DSBs) in degenerated colonic epithelium [99]. miR-23a and miR-155 in EVs can induce lamin B1-dependent replication fork collapse and inhibit homologous recombination (HR) by targeting the HR-regulator RAD51 [99]. The role of PMN-derived EVs in promoting DSB formation and suppressing DSB repair through the downregulation of lamin B1 and Rad51 was confirmed again in 2019 [100]. Furthermore, there is another explanation for PMN transepithelial migration. Butin-Israeli et al. showed that during transepithelial migration, the EVs derived from PMN were deposited on the IECs, leading to the loss of epithelial cadherins while enhancing the PMN recruitment [101]. Meanwhile, the other immune cell-derived EVs exhibited high immunomodulatory capacity to be attractive agents. EVs released by the granulocytic myeloid-derived suppressor cells caused a decrease in the proportion of Th1 cells and an increase in the proportion of regulatory T-cells in colitis mice [102]. WNT/*β*-catenin signaling, one of the major sources of WNT ligands [103, 104], is significant for intestinal homoeostasis and the intestinal epithelium. Macrophage-derived EVs can rescue the intestinal stem cells and enhance the survival rate of the enterocytes after radiation injury through the regulation of WNT function [105].

## 3. The Clinical Potential of EVs in IBD

As previously discussed, scientific interest in EVs has been stimulated due to their key roles in cell-cell and cell-organism communication. There is an urgent need to convert these fundamental achievements into clinical applications. Therefore, an increasing number of studies regarding EVs have been proposed to explore its role as a source of diagnostic and prognostic markers or as promising pharmaceutical vehicles.

### 3.1. Clinical Potential of EVs as Biomarkers

In recent years, multiple studies have investigated more precise markers of cancer. One essential approach aimed at diagnosing the development of cancer is based on the cargo of EVs. In 2014, Li et al. confirmed that using EVs was more amenable to the development of a disease marker panel rather than whole bile [106]. Likewise, a large body of work focusing on purifying EVs, increasing the abundance of cargo, and decreasing heterogeneity of the sample has been produced [107–109]. In 2018, a laboratory-built high-sensitivity flow cytometer was established for quantitative multiparameter analysis of single EVs. According to the corresponding report, the challenge of profiling and sizing the individual EVs was conquered through this new method. The author used this method to analyze blood samples from patients with colorectal cancer and healthy controls, and they obtained an accurate resolution and profile of EVs, thereby identifying CD147-positive EVs as a sensitive biomarker for colorectal cancer [110]. Similarly, miRNA in EVs can be an essential biomarker for the detection of disease recurrence. A previous study showed that the miR-17-92 cluster is highly expressed in microRNAs in patients with a poor prognosis [111]. IBD is known to potentially increase the risk of developing cancer [112–114]. However, there is a lack of promising biomarkers for the complicated surveillance of IBD. In 2015, Polytarchou et al. demonstrated that miR-214 is associated with the progression of IBD, and reducing its expression can slow the development of colitis and colitis-associated cancer in mice [115]. Interestingly, miR-214 also has been detected in the EVs of many gastroenterology cancers [116, 117]. In the meantime, isolating miRNAs from exosomes has been proven to be more stable and reliable than biomarkers in many studies [118–120]. These findings imply the function of EVs to monitor the cancer progression of IBD.

Circulating pathogenesis-related EVs have emerged as promising biomarkers to monitor disease development and as novel targets for future anti-inflammation therapies in IBD. In 2017, Zheng et al. investigated the high sensibility of salivary exosomal PSMA7 on IBD diagnosis [121]. This study identified the proteins within EVs by using a liquid chromatograph-mass spectrometer, and PSMA7 was shown to be associated with inflammation and immune response as well as depressive disorder in many studies [122, 123]. Rab proteins of the GTPase family are involved in selective packaging and docking at the plasma membranes of EVs [124]. With regard to the intestinal immune balance, the numbers of RAB27A- and RAB27B-positive immune cells increased in the colonic mucosa of patients with active ulcerative colitis (UC) compared to the healthy controls [125, 126]. Double knockdown of Rab27A and Rab27B led to interference in protecting mice from T-cell-transfer-induced colitis, which authenticated the crucial role of Rab27-mediated EVs in the treatment of IBD [127, 128]. All these findings indicate that EV biogenesis acts as a key strategy for the diagnosis and/or therapeutic potential of EVs in IBD.

### 3.2. The Clinical Potential of EVs on Treatment

Targeting specific cargo and transmembrane integrin of EVs might alleviate the inflammation of intestines. In the intestinal tract, the interaction between IEC and EVs is weaker in EpCAM-knockout mice. In the meantime, the protective effect of EVs has been decreased in IBD [76]. Genetic material within EVs shows its potential therapeutic role in IBD. Bone marrow mesenchymal stem cells (BMSCs) transfected with lentivirus to overexpress miR-200b can release EVs packaged with miRNA-200b. The miR-200b-EVs significantly suppressed ZEB1 and ZEB2 to reverse the morphology in TGF-*β*1-treated IEC-6 cells and ameliorate the TNBS- (2,4,6-trinitrobenzene sulfonic acid-) induced colon fibrosis histologically [129]. EVs secreted by mesenchymal stromal cells (MSCs) have been proposed as important mechanistic relievers in response to cellular inflammation through paracrine effects [130–132]. In addition, Harting et al. demonstrated that EVs from MSCs (MSC-EVs) stimulated with TNF-*α*+IFN-*γ* attenuated the release of proinflammatory cytokines in vitro [133]. Mao et al. proved the EVs derived from human MSCs can relieve the phenotypes of IBD in mice. After treatment with MSC-EVs in DSS-induced IBD mice, the expression of the IL-10 gene increased while those of the TNF-*α*, IL-1b, IL-6, iNOS, and IL-7 genes decreased in the colon tissues [134]. Additionally, Yang et al. confirmed the potential of BMSC-EVs in protecting the TNBS-induced colitis model via attenuating oxidative stress and apoptosis [135]. Generally speaking, IBD is caused by the breakdown of innate immunity and the aberrant activation of the immune system. Therefore, it is consequently conceivable that EVs from the immune cells may be used as a new therapeutic intervention of IBD. As mentioned before, EVs derived from DCs can relieve the progress of disease via immune-stimulatory or immune-suppressive effects. Meanwhile, some conditioned DCs secreted EVs to make progress against the IBD [97]. The recent wave of research on EVs assists in the exploration of the utilization of artificial nanoparticles in disease treatment. A considerable amount of work has been performed regarding IBD treatment with EV-like nanoparticles. In 2019, Han et al. expanded the use of bioadhesive chitosan materials on colloidal-stable nanotherapeutics. This exhibited safe and precise accumulation to local diseased lesions in the gastrointestinal tract [136]. In 2018, Zahra et al. utilized intestinal organoids as carriers of 5-ASA-loaded poly nanoparticles to alleviate IBD [137]. Similarly, Bo et al. used mannosylated bioreducible cationic polymers to synthesize RNA interference nanoparticles to reduce cytotoxicity and promote treatment effectiveness in IBD [138]. While innately derived from cells and microbiota, EVs are much more biocompatible and stable when compared with nanoparticles. What is more, EVs could also be engineered, thus indicating the therapeutic role in disease such as IBD [139, 140].

Breast milk not only is rich in nutrition but also provides a diverse array of microbiota and immunoglobulin. It may shape the neonate gut immune system actively and convert it toward a mature immune system capable of responding appropriately to encountered antigens [141–143]. EVs in milk are one of the most recently identified components that may influence intestinal homeostasis. Therefore, the discovery ten years ago that breast milk contains abundant immune modulatory EVs has earned plenty of attention in this field of study [144]. Additionally, breast milk EVs containing genetic material and proteins delivered to infant mucosae offer novel insights into the mechanisms of action for drug delivery in the intestinal tract. EVs are quite stable even in simulated gastric/pancreatic digestion [145] so that EV microRNAs in human breast milk can be delivered to the intestinal epithelia of infants [146]. Soon after, Liao et al. illustrated that milk-derived EVs enter human intestinal crypt-like cells, suggesting the possibility of EVs from breast milk altering the neonatal mucosal conditions [147]. Several studies have reported that treatment with milk EVs can significantly increase IEC viability, proliferation, and stem cell activity [148–150]. Breast milk reduces the incidence of necrotizing enterocolitis (NEC), and EVs in breast milk offer a new path in the mechanism for breast milk attenuating cell death in intestinal epithelial cells, as well as the possibility of transporting drugs in milk [151–154].

siRNA has a potential therapeutic effect but has various physiological limitations, including unstable delivery. Using lipofection to encapsulate AF488 in milk whey EVs guarantees their internalization by Caco-2 cells [155]. Recently, protein within EVs has been the subject of intense research, and one such intestinal EV-containing molecule is TGF-*β*1. Intestines produce EVs containing high levels of TGF-*β*1 that can alleviate the severity of IBD by inducing regulatory T-cells and immunosuppressive dendritic cells in DSS-induced IBD mice [76]. Meanwhile, the endogenous molecule annexin A1 (ANXA1) has been reported to promote epithelial restitution in a colitis-induced mucosal damage model. In signaling via binging to formyl peptide receptors (FPRs), epithelial cells release the potent endogenous mediator ANXA1 as a component of EVs that promotes the repair of intestinal mucosal inflammation. Leoni et al. also observed the increased concentration of ANXA1 through EVs in the sera of patients with IBD and found that it correlated with disease severity [81]. Additionally, this correlation could conduce to EVs emerging as promising biomarkers not only to monitor IBD progression but also to have potential effects in future therapies. In fact, an in vivo proof of the study regarding ANXA1 proved that encapsulated targeted polymeric nanoparticles (Ac2-26 Col IV NPs) accelerated the recovery of intestinal inflammation in experimental IBD mice [81]. Similarly, nanoparticles, artificial EVs loaded with rifaximin, have high encapsulation efficiency, relatively high loading capacity, and a predetermined in vitro release profile [156, 157]. These studies regarding cargo manipulation suggest that EVs may be beneficial as drug delivery vehicles. So far, relevant EV studies considering practical clinical applications are usually preclinical studies based on animal or cell models. This indicates that further studies are required to explore the application prospects in clinical settings. However, such research is still in its infancy and should not be underestimated, whether in diagnosis or treatment.

## 4. Conclusion

**Figure 2:**
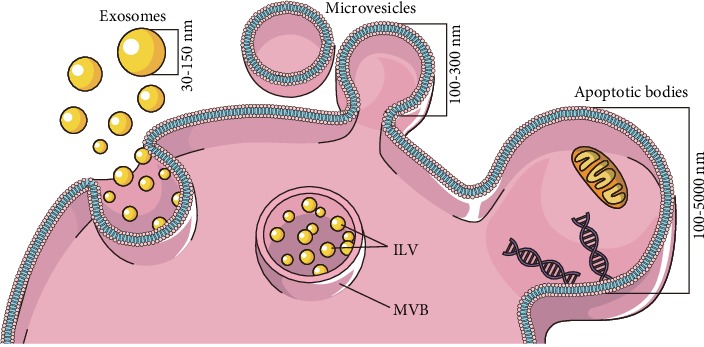
The interaction between bacteria, immune cells, and intestinal cells through EVs in gut. The schematic depicts the pathways by which OMVs derived from the member of microbiota take part in the hemostasis of intestines through various pathways. Bacteroides in virtue of HtrA packing in OMVs can facilitate its transmigration across polarized intestinal epithelial cells through the cleavage of the TJ and AJ. EVs from pathogenic bacteria can stimulate the production of interleukin-8 (IL-8) in IECs via the TLR5 and TLR4/MD-2 complex signaling pathway. OMVs from commensal bacteria containing peptidoglycans could colocalize with NOD1, trigger the NOD1 signaling pathway, and improve the expression of NF-*κ*B, IL-6, and IL-8. EVs derived from bacteria on benefits of maintaining intestinal hemostasis reflected in increasing ZO-1 and claudin-14, decreasing claudin-2 in probiotic, and reducing the expression of IL-6 and TLR2-dependent EV internalization by DCs. EVs from IECs carrying *αβ*6 activate LTGF*β* in intestinal tolerogenic DCs and Tregs. M*ϕ*-derived EVs can enhance survival of enterocyte through WNT function. Note: OMVs: out membrane vesicles; HtrA: high-temperature requirement A; TJ: tight junction; AJ: adherens junctions; GJ: gap junction; TLR4/5: Toll-like receptor; IL-8: interleukin 8; IL-6: interleukin 6; M*ϕ*: macrophages; EVs: extracellular vesicles; TGF-*β*: transforming growth factor-*β*; NOD1: Nucleotide Binding Oligomerization Domain Containing 1; IECs: intestinal epithelial cells; LTGF*β*: latent transforming growth factor-*β*; TGF-*β*: transforming growth factor-*β*; DCs: dendritic cells; Tregs: T regulatory cells; WNT: wingless/integrated.

In the current review, we discussed the source (Table 1), cargo, and origin of EVs and their roles in the pathogenesis and progression of IBD. We mainly focused on EVs from microbiota and enterocytes to clarify the relationships among EVs, microbiota, and intestinal inflammation (Figure 2). In addition, the clinical potential of EVs as biomarkers and their therapeutic effects on IBD were summarized.

## Figures and Tables

**Table 1:** Various source of EVs related to IBD.

Source	Mechanism	Reference	
Stem cell	Alternating COX2/PGE2 pathway	[133]	MSC
	Inhabiting iNOS and IL-7 pathway	[134]	MSC
	Attenuating oxidative stress and apoptosis pathway	[135]	BMSC
	Inhibiting EMT by targeting ZEB1 and ZEB2	[129]	BMCS
Milk	Stimulate intestinal stem cell activity	[148]	Breast milk
	Activating the hypoxia-inducible factor signaling pathway	[149]	Yak milk
	Inhibiting P53 pathway	[150]	Porcine milk
	Inhibiting oxidative stress pathway	[152]	Breast milk
Immune cell	Inhibiting Th1 cells proliferation and promoting Treg expansion	[102]	Myeloid-derived suppressor cells (MDSC)
	WNT/*β*-catenin signaling	[51, 105]	Macrophage
	Inducing Th1 polarized CD4+ T-cells	[93, 94]	Dendritic cells
	Enhancing the inflammation response via proinflammatory microRNAs and MPO	[98–101]	Neutrophil
Microorganism	Eliciting the release of proinflammatory IL-8	[51]	Escherichia coli C25
	Regulating ZO-1 and ZO-2	[53]	Escherichia coli Nissle 1917
	Promoting intracellular Ca^(2+)^ signaling	[54]	Bacteroides thetaiotaomicron (BtMinpp)
	Ameliorating the production of IL-6	[55]	Akkermansia muciniphila

COX2: cyclooxygenase 2; PGE2: prostaglandin E2; iNOS: inducible nitric oxide synthase; IL-7: interleukin 7; EMT: epithelial-mesenchymal transition; ZEB1: zinc finger E-box binding protein 1; Th1: T helper cell; Tregs: T regulatory cells; WNT: wingless/integrated; IL-8: interleukin 8; ZO-1: zonula occluden-1; IL-6: interleukin 6; MSC: mesenchymal stem cell; BMSC: bone mesenchymal stem cell; MDSC: myeloid-derived suppressor cells; BtMinpp: Bacteroides thetaiotaomicron.
